# Image-Based Polygonal Lattices for Mechanical Modeling of Biological Materials: 2D Demonstrations

**DOI:** 10.1021/acsbiomaterials.0c01772

**Published:** 2021-06-01

**Authors:** Di Liu, Chao Chen, Teng Zhang

**Affiliations:** † Department of Mechanical and Aerospace Engineering, Syracuse University, Syracuse, New York 13244, United States; ‡ BioInspired Syracuse, Syracuse University, Syracuse, New York 13244, United States

**Keywords:** polygonal lattice model, image-based simulation, biomaterials, biomimetic composites

## Abstract

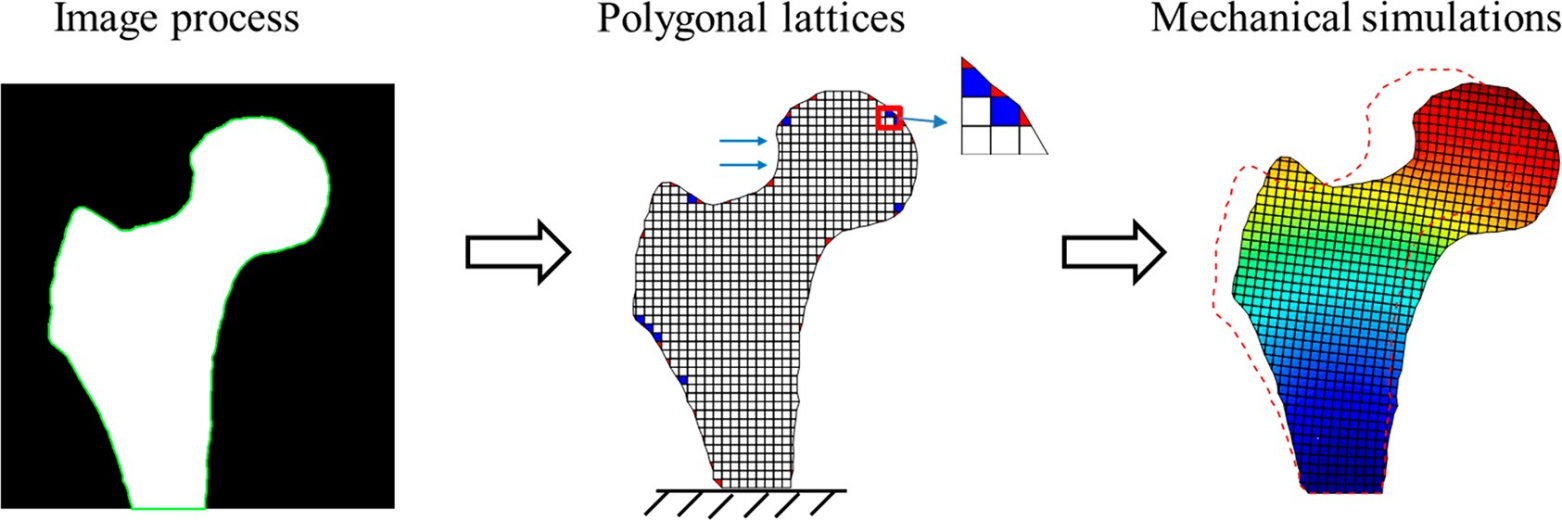

Understanding the structure–property relationship of biological materials, such as bones, teeth, cells, and biofilms, is critical for diagnosing diseases and developing bioinspired materials and structures. The intrinsic multiphase heterogeneity with interfaces places great challenges for mechanical modeling. Here, we develop an image-based polygonal lattice model for simulating the mechanical deformation of biological materials with complicated shapes and interfaces. The proposed lattice model maintains the uniform meshes inside the homogeneous phases and restricts the irregular polygonal meshes near the boundaries or interfaces. This approach significantly simplifies the mesh generation from images of biological structures with complicated geometries. The conventional finite element simulations validate this polygonal lattice model. We further demonstrate that the image-based polygonal lattices generate meshes from images of composite structures with multiple inclusions and capture the nonlinear mechanical deformation. We conclude the paper by highlighting a few future research directions that will benefit from the functionalities of polygonal lattice modeling.

## 1 Introduction

Mechanical modeling plays a crucial role in understanding organisms’ physiological responses from the cellular level^1^ to the whole-body level^2^ under complicated in vivo environments. Various biological structures and materials, such as bones, cartilage, and skin, bear and respond to mechanical loads. In addition, mechanical modeling provides a powerful tool for understanding, diagnosing, and treating certain diseases and injuries. For instance, the local mechanical property changes compared to the normal tissue are pathological indications of cancers,^3^ and a short cervix’s lack of mechanical support is related to preterm birth.^4,5^ Because of the complexities in biological environments, successful progress of analyzing mechanical properties of biological structures relies on the development of quantitative computational modeling with high fidelity. One of the prominent complexities is the complicated anatomy with heterogeneous material phases in various organs and tissues, such as bones,^6−10^ brains,^11^ and cartilages.^12^ It has been well documented that structure heterogeneity plays a critical role in determining the mechanical properties of biological materials.^13,14^ The significance of heterogeneity also shows in biomimetic materials with similar heterogeneous structures.^15−17^ The interaction between different phases requires coupling across materials with distinctive rheological properties through interfaces. Handling these complexities in modeling poses great challenges for computational science and engineering and has attracted attention to developing more efficient modeling algorithms and simulation platforms.^18−23^

Image-based analysis is an important tool for understanding the mechanical functions of biological materials.^24−32^ The image analysis extracts accurate shape boundaries and material interfaces from images, which serve as the inputs for proper mechanical simulations. The image-based analysis significantly benefits from many new technologies^33−35^ that reveal the detailed features of biological materials, such as scanning electron microscopy (SEM),^36^ X-ray computed tomography (XCT),^37^ and dual-energy X-ray absorptiometry (DXA).^38^ For example, high-resolution magnetic resonance (MR) head images have enabled personalized numerical simulations of the mechanical response of brains under impact^39−41^ or decompressive craniectomy.^42^ DXA-based patient-specific finite element simulations can analyze the stress state of hips and assess the fracture risk.^43−45^ The major advantage of the image-based mechanical analysis is the convenience of utilizing the native uniform meshes from image pixels.^46^ Meanwhile, irregular boundaries suffer from staggered elements. Conventional simulations employ very fine meshes to ensure accuracy. Recent advancement along this direction has shown that adaptive isogeometric analysis^47^ can achieve a balance between the computation complexity and simulation accuracies at boundaries. To improve the description of the boundaries, nonuniform and conformal meshes were adopted to describe the complicated boundaries. Because of the simplicity and generality, triangle and tetrahedron elements have been widely used for these nonuniform meshes in two-dimensional (2D) and three-dimensional (3D) analyses,^48−52^ respectively. However, these elements usually require higher-order interpolation functions to capture the nearly incompressible deformations. Besides, a combination of uniform interior meshes and conforming meshes at boundaries has attracted tremendous attention in the last decades. The mesh combination is achieved through enriched elements, such as X-FEM^53^ and scaled boundary elements,^54−57^ to follow the boundaries. The latter method has advantages in both mesh generation and mechanical simulations but mostly focuses on small deformations.

Despite intensive studies and significant progress, there is still a lack of a simple and versatile simulation platform to process images and perform mechanical analysis with the capabilities of handling nonlinear deformation and nearly incompressible materials. Here, we aim to establish a simple and efficient lattice modeling platform with image-based polygonal meshes. Lattice models compute the elastic deformation energy using springs^58,59^ associated with the distance change between vertices. It has been widely used in bones and bone-inspired materials,^60,61^ porous materials,^62^ multiphysics gels,^63^ and soft magnetic materials.^64^ For example, triangle lattice-spring models have been shown to successfully capture the complicated fracture in hierarchical bioinspired composites.^65−68^ Compared to the existing lattice-spring model that usually has a fixed Poisson’s ratio because of its nature of two-point interaction, our recent work on a quadrilateral 2D lattice model^69^ showed that an additional strain energy term associated with the area change can be integrated to the model represent the volumetric strain to capture different Poisson’s effects. In addition, the quadrilateral 2D lattice model is isotropic, unlike most lattice-spring models that exhibit anisotropic behaviors based on the lattice architecture. In this work, we extend the regular quadrilateral lattices to general polygonal lattices. By introducing irregular polygons, we extend the modeling capability to discretize the complicated geometry at the interfaces and boundaries. Meanwhile, this method maintains the uniform square background meshes. This work focuses on 2D structures with idealized scenarios, and the design of the computational algorithms can be extended to the general 3D structures in future works and are anticipated to investigate the real-world biomechanical responses. The simplicity of the polygonal lattice model makes it a very promising candidate to be integrated with existing simulation methods to tackle multiphysics problems, such as coupling fluid and structure such as the lattice Boltzmann method for fluid–structure interactions.^70^

## 2 Image-Based Polygonal Lattice Model Analysis

In this section, we illustrate the idea of the image-based polygonal lattice model analysis. Without losing generality, we take an example of the mechanical simulation of a 2D proximal femur, whose deformation and fracture have been the benchmarks to examine modeling methods including the image-based finite element simulations.^43−45^ Here, we present a method of maintaining uniform meshes in the majority of the structure, with nonuniform polygonal meshes limited near the boundaries.

**Figure 1:**
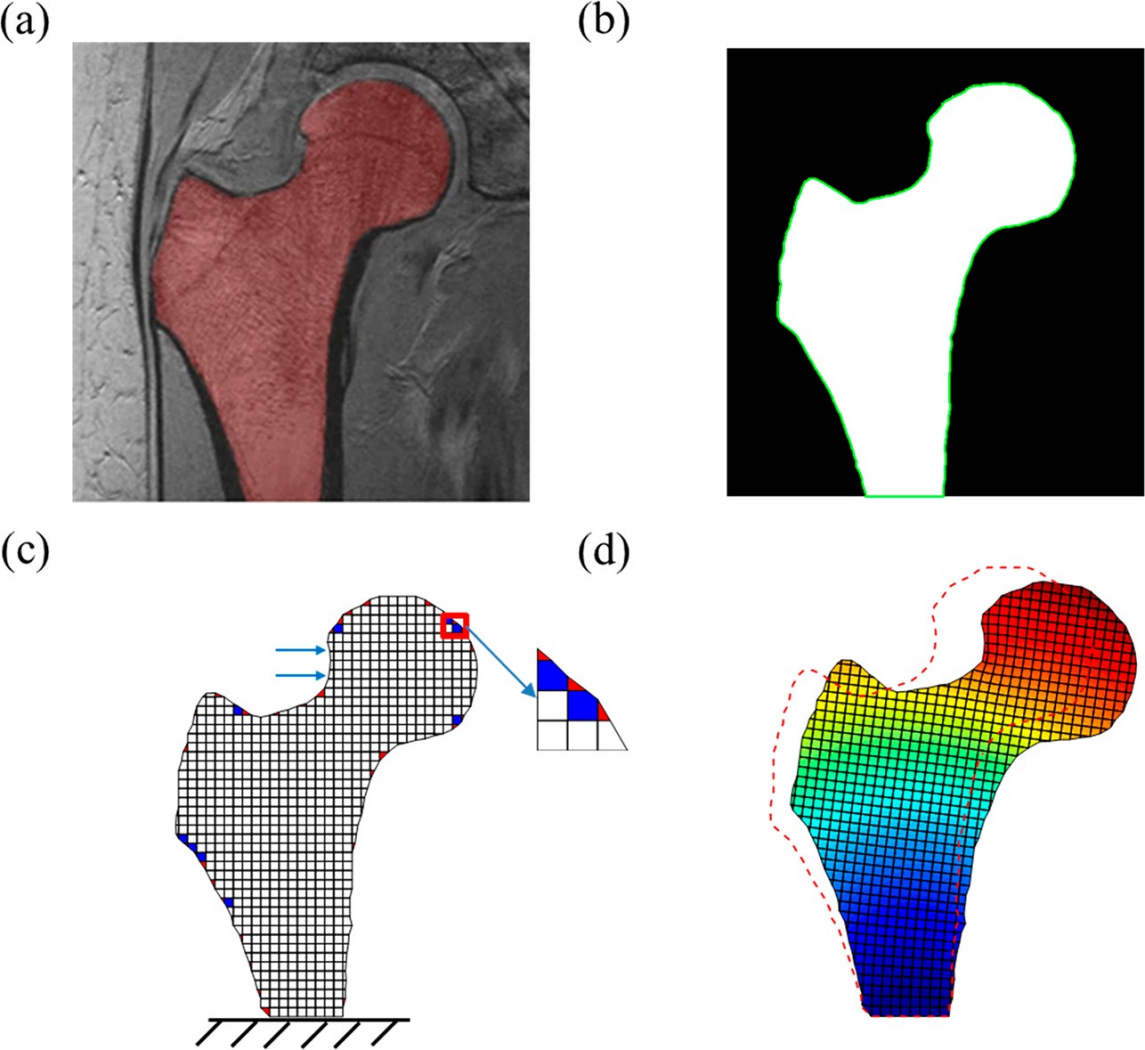
Procedure of a polygonal lattice modeling. (a) Magnetic resonance (MR) femur image and its segmentation. The image data was obtained from ref (71). (b) Image process of extracting the boundary. (c) Directly splitting of the boundary structured meshes to form polygonal lattices. The inner part maintains the structured square meshes. (d) Bone bending simulations with the polygonal lattice model. The initial bone state is denoted as the red dash line.

We first import an image after segmentation of magnetic resonance (MR) image^71^ (Figure 1a) to *MATLAB*. We identify the proximal femur boundary (Figure 1b) on the binary image. The boundary nodes of the proximal femur form a polygon with small edges. The polygon region is then created on a binary image with a desired resolution to generate uniform and larger meshes inside the proximal femur (Figure 1c). The intersects of the polygons and the background uniform meshes are further identified to split the boundary squares into polygon pairs (Figure 1c), such as a pair of a triangle and a pentagon, or two quadrilaterals. In principle, this method accommodates general polygons, such as hexagon and heptagon. These combinations will be considered in future studies. After the element split, we set up the polygonal lattice model by computing the spring constants inside each polygon. In the last step, we demonstrate the bending deformation of the proximal femur through the polygonal lattice modeling (Figure 1d).

The critical step is modeling the polygonal lattices, which was not discussed in our previous work on the lattice models. Similar to the quadrilateral lattice model, we show that the polygonal lattice model can be established by applying the technique of computing the lattice spring constant from the shape functions in polygon element methods,^72−76^ also known as virtual elements. We choose the neo-Hookean material model for the material’s constitutive law, which accounts for the geometric nonlinear deformations and moderate stretching strains (e.g., 30% for PDMS^77^). Consider the coordinates of a material point in an undeformed reference state **X.** The coordinates of the same material point after deformation are denoted as **x**. The deformation gradient **F** = ∂**x**/∂**X**. The strain energy density function *U* is
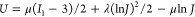
1where μ is the shear modulus, λ is the Lamé constant, *I*_1_ is the first invariant of the right Cauchy-Green deformation tensor **F**^T^**F**, and *J* is the determinant of the deformation gradient **F** that represents the volume dilation.

Following our previous work on the quadrilateral lattice model,^69^ we express the strain energy inside a polygon into the stretching energy *U*_*I*_1__ associated with the distance change of polygon vertices and the energy *U*_*J*_ associated with the volume change. In our current focus of the 2D plane-strain condition, the volume change is simplified as the area change of polygons, and the volumetric term *U*_*J*_ can be written as
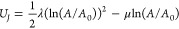
2where *A* and *A*_0_ represent the areas of the polygon at the deformed and reference configurations, respectively. The same formula has been shown to avoid volumetric locking for nearly incompressible materials,^69^ especially for polygons with more than three edges.

**Figure 2:**
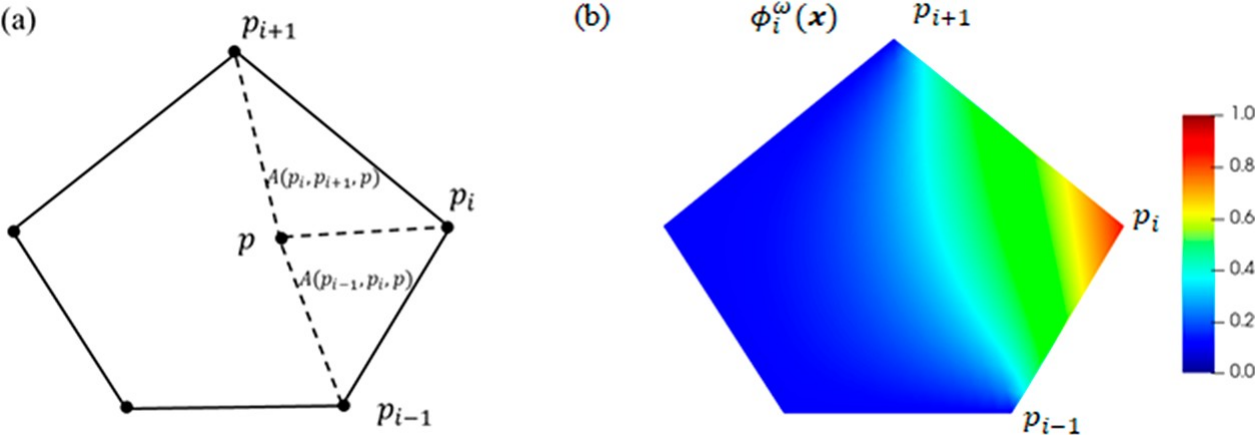
Shape functions of a general polygon (a pentagon as a representative case). (a) Illustration of nodes related to the Wachspress’s basis functions in a pentagon. (b) Shape function of the *i*-th node in a pentagon.

To find the lattice spring constants in the energy term of *U*_*I*_1__, we first express the deformation gradient with the nodal coordinates
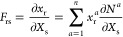
3where *N*^*a*^ is the shape function, *r* and *s* are the coordinate system indices, and *x*_*r*_^*a*^ is the nodal coordinate in the deformed state. Here we focus on convex polygons and construct the shape function of the polygonal element with the Wachspress method.^76^ Wachspress shape functions (ϕ_*i*_^ω^) are constructed through the barycentric coordinates, such that *N*^*i*^ = ϕ_*i*_^ω^. The Wachspress’s basis functions on a polygonal element with *n* sides are expressed as^76^
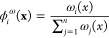
4
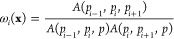
5where ω_*i*_(**x**) is the weight function of the *i*-th node *p*_*i*_, node *p* is a Barycentric node, *A*(*l,m,n*) is the signed area of the triangle [*l m n*] as shown in Figure 2a. Figure 2b shows the contour of a representative shape function. The gradient of the Wachspress shape function can be written as
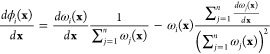
6where
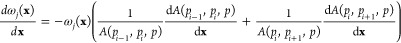
and **x** is the coordinate of node p.

With the 2D plane-strain problems, the strain energy associated with *I*_1_ inside the element is
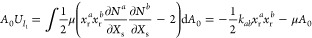
7where . Here the dummy indices *r* and *s* take the summation of 1 and 2. Equation 7 shows that *U*_*I*_1__ is a quadratic function of nodal positions. By leveraging partition of the unity of the polygonal element shape functions (i.e., ), we rewrite the strain energy as the summation of energies in springs that link different nodes in the lattice structure,
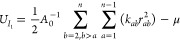
8where *r*_*ab*_ = *x*^*a*^ – *x*^*b*^, *a* = 1, 2, ..., *n* and *b* = 2, 3, ..., *n* represent lattice springs between nodes *a* and *b*, and *k*_*ab*_ is the corresponding spring stiffness. This can be seen as a generalization of the quadrilateral lattice model where the node number can only be 4.

## 3 Benchmark Tests

Before applying the polygonal lattice model to the image-based numerical analysis, we perform benchmark simulations to validate the model. We first verify a pure-shear tension test and a cook membrane test on homogeneous samples. The initial polygonal mesh is generated by the *MATLAB* package “PolyMesher”.^78^ Next, we examine the capability of modeling composite structures with a circular inclusion and a sinusoidal interface. The simulations adopt the following unit system: mm for length, mN for force, and kPa for stress and modulus.

**Figure 3:**
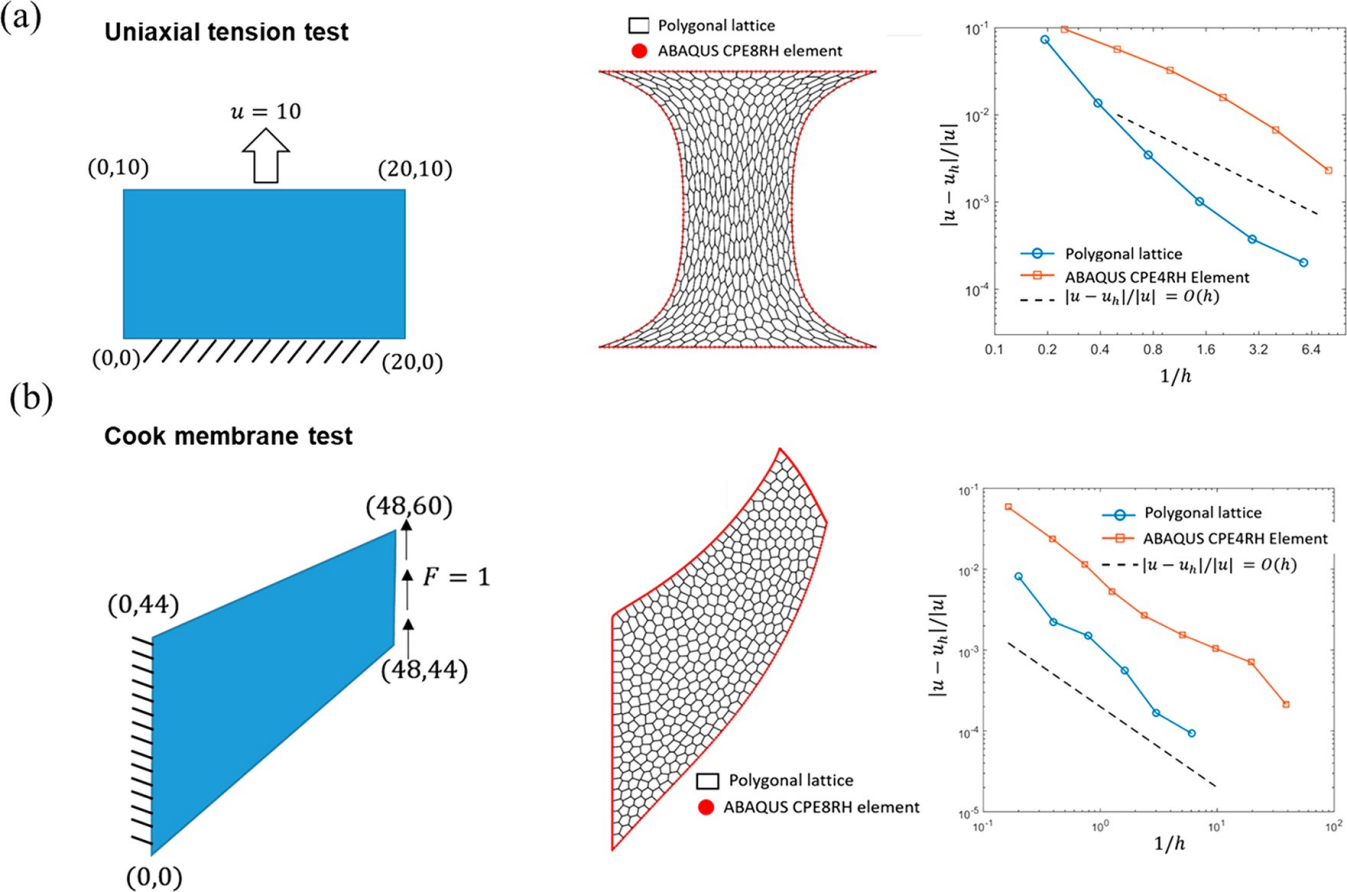
Benchmark tests for completely polygonal lattices. (a) Uniaxial tension and (b) cook membrane tests on homogeneous samples. The polygonal lattice model results are compared to the FEM results (red curves) for validation. For the convergence rate plots, the reference solutions “u” are *ABAQUS* simulations based on fine and second-order element (CPE8RH).

For the uniaxial tension test, we implement the polygonal lattice modeling on a rectangle with a width of 20 mm and a height of 10 mm. We adopt the Dirichlet boundary condition on the top and bottom layers, where the vertical displacement of the top boundary is 10 mm, and the bottom boundary is fixed as static (Figure 3a). For the cook membrane test, we prescribe uniform vertical shear traction of 1 kPa on the right boundary and fix the left boundary (Figure 3b). The material of these two test sets is nearly incompressible neo-Hookean material with a shear modulus of 1 kPa and a bulk modulus of 100 kPa. A finite element method platform, *ABAQUS*, performed the same simulation setting with 8-node hybrid elements (CPE8RH) for validation. Figure 3 shows that the simulation results of the polygonal lattice modeling accurately agree with that of the hybrid finite element modeling. In both cases, the polygonal lattice model gives a first-order convergence rate, like the quadrilateral finite element method (CPE4RH) in *ABAQUS* (Figure 3).

**Figure 4:**
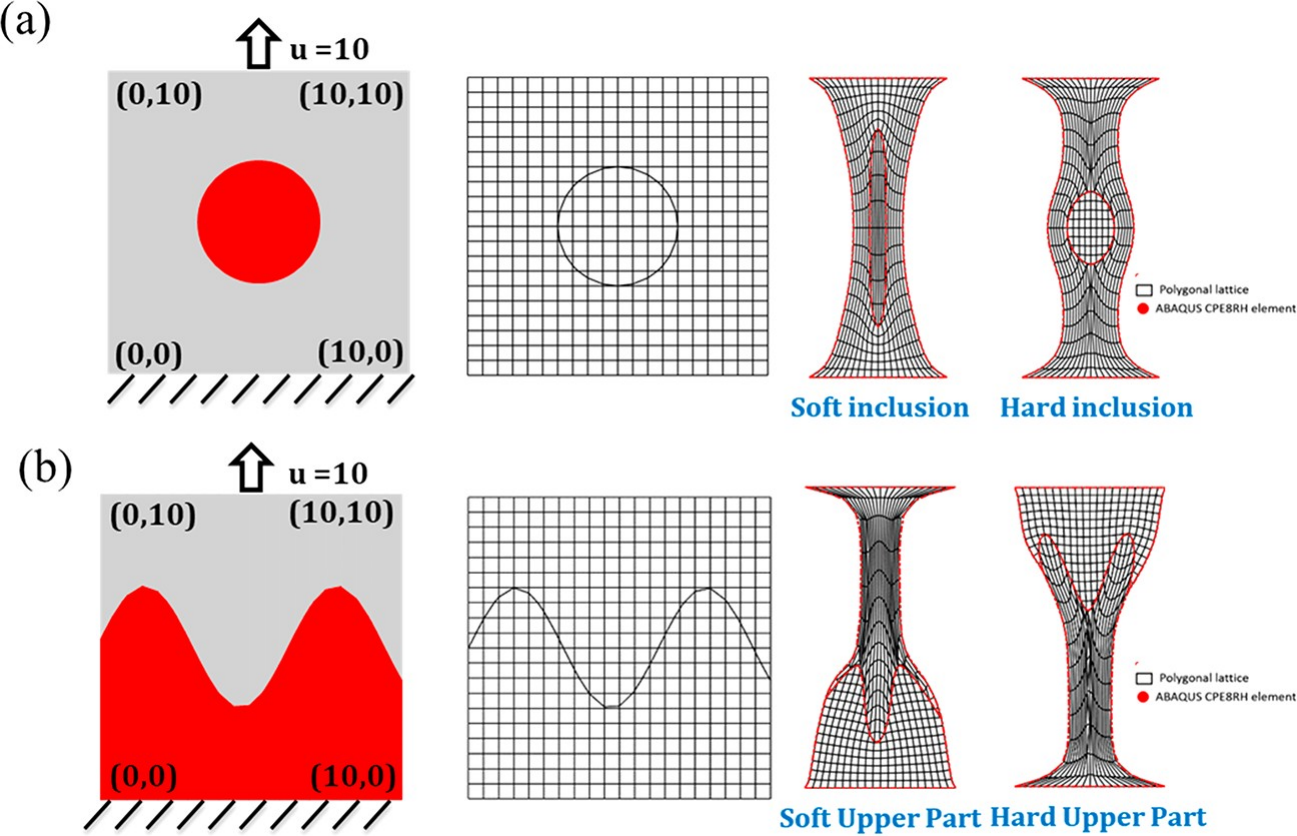
Heterogeneous composites with (a) a circular inclusion and (b) a sinusoidal interface. The polygonal lattice model results are compared to the FEM results (red curves) for validation.

We next apply the polygonal lattice model to two heterogeneous composites with a circular inclusion and a sinusoidal interface, respectively, where only the interfaces are modeled as polygons. For the sample with a circular inclusion, we perform two different sets of simulations with soft and hard inclusions. The matrix material is of the shear modulus 1 kPa and the bulk modulus of 100 kPa for both cases. For the soft inclusion, the shear modulus is 0.1 kPa, and the bulk modulus is 10 kPa, whereas for the hard inclusion, the shear modulus is 10 kPa and the bulk modulus is 1000 kPa. We apply the boundary condition with the tension displacement of 10 mm on the sample’s top layer and with a fixed bottom surface. Also, the same test set is imported into ABAQUS for FEM simulation using the CPE8RH element. We compared the results from the polygonal lattice model and the FEM simulations for validation (Figure 4a), which agree with each other well.

For the bilayer structure with a sinusoidal interface, we assign different material properties on the part above the sinusoidal interface and the part under it. Two sets of tests are implemented with a soft upper part and hard upper part, respectively. For the lower part of both simulation sets, the shear modulus is 1 kPa, and the bulk modulus is 100 kPa. As regarding the soft upper part is of the shear modulus 0.1 kPa and the bulk modulus 10 kPa. The shear modulus of the hard upper part is 10 kPa, and the bulk modulus is 1000 kPa. The polygonal lattice model matches well with the results from FEM simulations, as shown in Figure 4b.

## 4 Tension and Shear Deformation of Composite Materials

**Figure 5:**
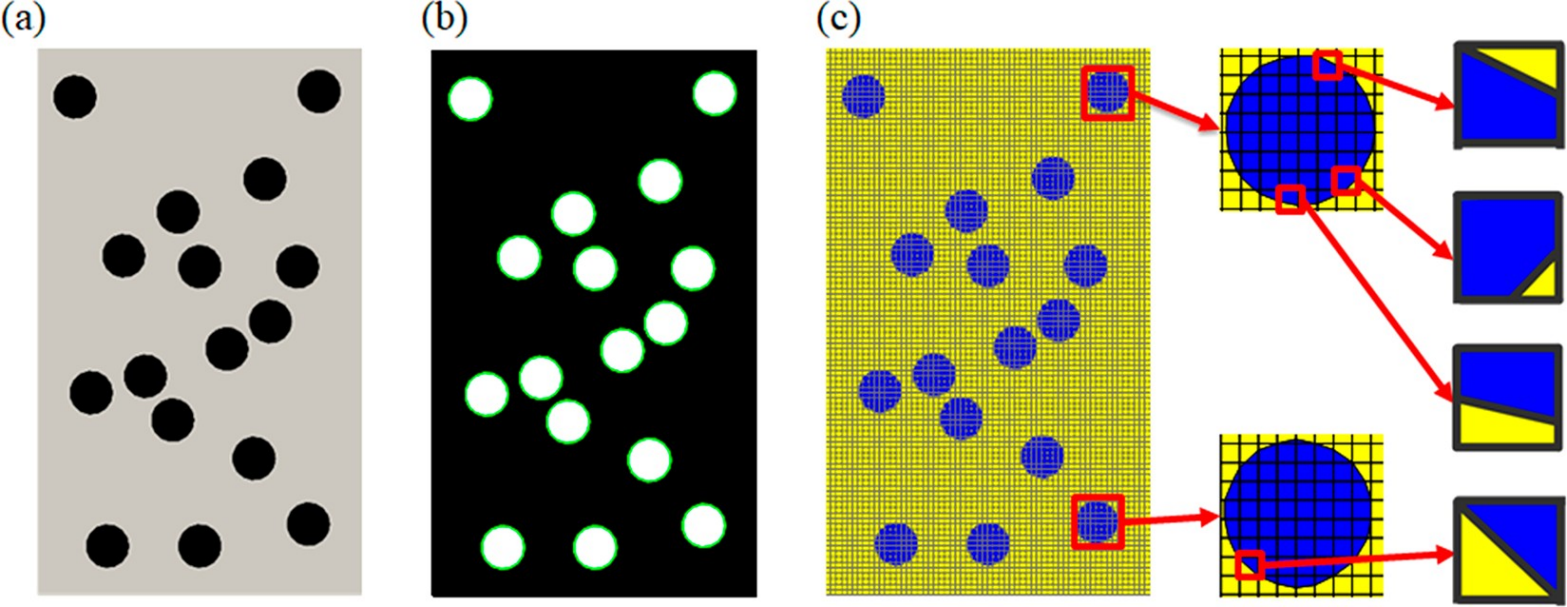
Polygonal mesh processing from a composite with multiple inclusions. (a) Original composite image. (b) Inclusion boundary identification. (c) Structured meshes with polygons at the inclusion–matrix interfaces.

Many biological materials exhibit heterogeneous microstructures, such as bones,^7^ bamboos,^32^ and biofilms,^79^ which are regarded as composites with inclusions embedded in matrices. Similar composite models also describe the distributed tumors in organs,^80^ where the tumor tissues are inclusions with different mechanical properties from their surrounding tissues. Therefore, it is important to understand the structure–property relationships of these composite structures. As a model system, we apply the polygonal lattices to a composite sample with 16 circular inclusions. The image of the sample as the initial input is shown in Figure 5a. The 2D sample is a rectangle of a width of 60 mm and a height of 100 mm. The inclusions are circles with a radius of 4 mm.

To generate the polygonal lattice for the composite, we first use *MATLAB* image processing functions to obtain coordinates of the boundary nodes (Figure 5b). We then find the intersects between structured elements and the inclusion interfaces. We label the elements that are fully outside or inside the inclusions. We divide any element cut by the inclusion boundaries into two polygons, where one polygon is inside the inclusion and the other outside. There are four cases of cut elements: a triangular element and a quadrilateral element, a triangular element and a pentagonal element, two quadrilateral elements, and two triangular elements (Figure 5c). For structures with complex interfaces, topological ambiguity can happen at the intersected elements, requiring special treatments to obtain high-quality mesh.^81,82^ As a demonstration of the proposed model, we adapt a fine background mesh for the current simulations and merge the intersection point between the interface and the regular mesh with the vertex (e.g., 0.01 of the lattice length) to remedy this issue. More studies are required to explore the mesh sensitivity and adaptive meshing techniques in the future, which are important to handle regions with complicated interfaces and high curvatures.

Different material domains, such as the inclusion and matrix, can be identified and labeled from the color values of the image. We then assign material properties to the elements inside or outside the inclusions. Here we include two sets of inclusion properties in the modeling. One is the composite with hard inclusions comparing to the matrix, and the other with soft inclusions. The shear modulus for the matrix part is 1 kPa, where the shear modulus of the soft inclusion is one-tenth of the matrix, and ten times for the hard inclusion.

**Figure 6:**
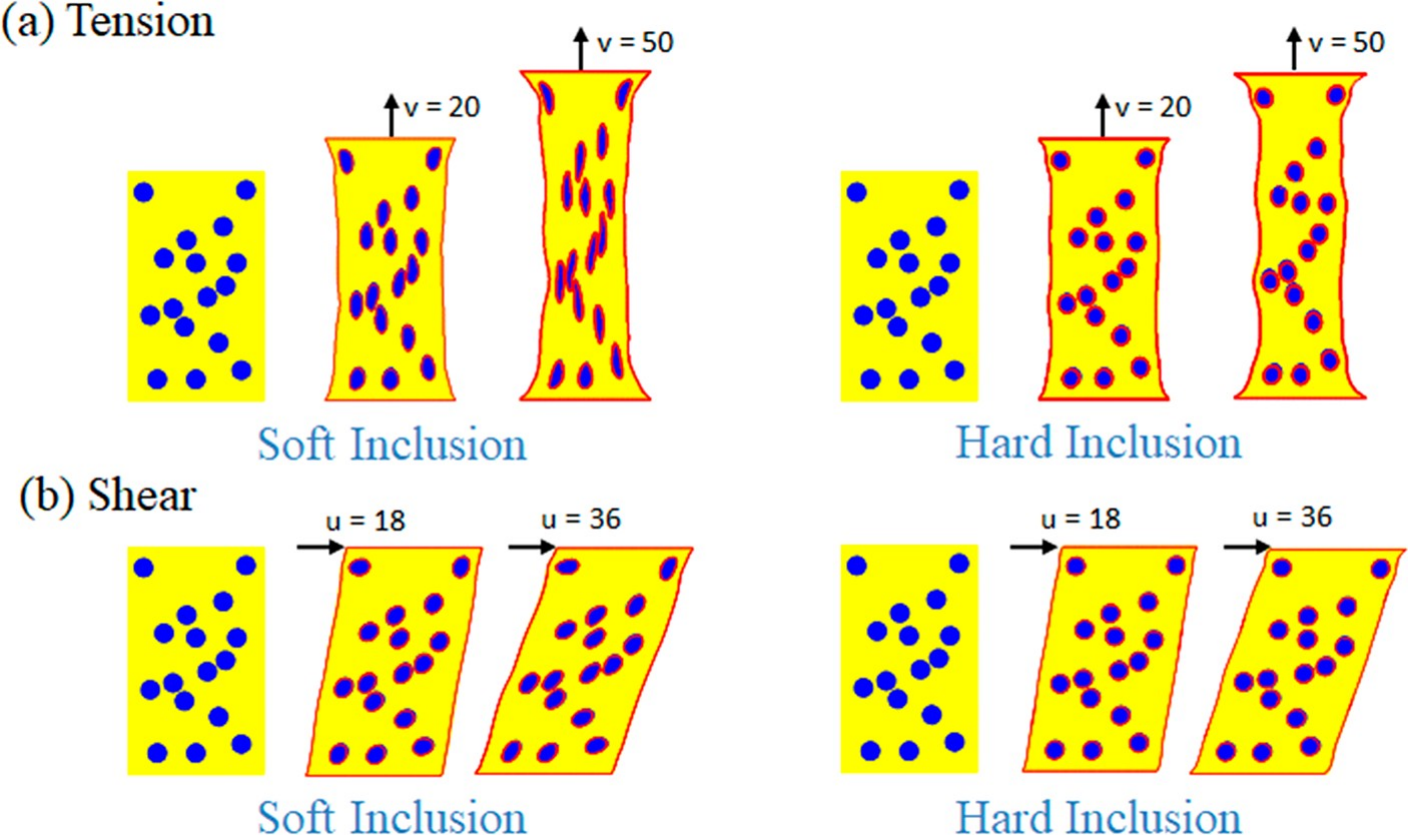
(a) Tension tests and (b) shear tests simulations of composites with multiple inclusions. The inclusions are either softer than the matrix or harder. The polygonal lattice modeling results are compared with FEM (red curves) for validation.

First, we conduct tension tests for both soft and hard inclusion cases. We fix the bottom layer and apply tension displacement on the top boundary. The results with the top displacement of 20 and 50 mm are in Figure 6a. We also perform the shear tests by fixing the bottom layer and applying a shear displacement on the top layer. The results with the shear displacement of 18 and 36 mm are in Figure 6b. All these four simulations are in good agreement with the ABAQUS results shown in the red lines.

## 5 Conclusion and Discussion

We presented an image-based polygonal lattice model for simulating the 2D plane-strain mechanical deformation of biological materials with complicated shapes or components. In the polygonal lattice model, the strain energy of a neo-Hookean solid comprises the stretching energy associated with the distance change of the vertices and the volumetric energy related to the area change of the polygon. The polygonal lattice further enables us to maintain uniform meshes inside homogeneous regions of the biological materials and only remesh the boundaries or material interfaces. The combination of meshes significantly simplifies the mesh generation from images for structures with complicated geometries that widely exist in biological materials and structures as well as bioinspired materials and structures. We performed benchmark tests to verify the proposed polygonal lattice models by comparing results with conventional FEM simulations. We further demonstrated its capability to generate meshes from images of composite structures with multiple inclusions and capture the nonlinear mechanical deformation.

The polygonal lattices model bridges the intuitive physical model of springs to the strain energy in continuum mechanics. We anticipate that this simple physical picture of the polygonal lattice lowers the technical barrier for researchers with diverse backgrounds to simulate the mechanical deformations of various biological and biomimic materials and structures. Although the current work reports promising results, a number of topics require further investigations. For example, developing a 3D polyhedral lattice model is demanded to model the real-world 3D biological and biomimic complicated structures. The formula proposed in the current paper works only for convex polygons and may lead to diverged simulations due to the loss of convexity. Therefore, it is of great interest in extending the current analysis into concave polygons. Adaptive meshing is another important technique to be investigated to further improve the efficiency of the model, especially for highly complicated structures under large deformation. More research efforts are needed to go beyond the neo-Hookean material model to incorporate more complex and realistic biological material models, such as the Fung-elastic material model^83^ and the Holzapfel-Gasser-Ogden constitutive model.^84^
